# No Evidence of Sexual Risk Compensation Following PrEP Initiation Among Heterosexual HIV Serodiscordant Couples in Kenya and Uganda

**DOI:** 10.1007/s10461-019-02720-4

**Published:** 2019-11-06

**Authors:** Katrina F. Ortblad, Randy M. Stalter, Elizabeth A. Bukusi, Kenneth Ngure, Andrew Mujugura, Connie Celum, Jared M. Baeten, Renee Heffron

**Affiliations:** 1 Department of Global Health, University of Washington, 908 Jefferson St, 12th floor, Seattle, WA 98104 USA; 2 Department of Epidemiology, University of Washington, Seattle, USA; 3 Department of Obstetrics and Gynecology, University of Washington, Seattle, USA; 4 Centre for Microbiology Research, Kenya Medical Research Institute, Nairobi, Kenya; 5 Department of Community Health, Jomo Kenyatta University of Agriculture and Technology, Nairobi, Kenya; 6 Infectious Diseases Institute, Makerere University, Kampala, Uganda; 7 Department of Medicine, University of Washington, Seattle, USA

**Keywords:** PrEP, Sexual risk compensation, Condom use, HIV, Serodiscordant couples, Africa

## Abstract

Recent studies among men who have sex with men suggest that sexual behaviors associated with risk of sexually transmitted infections increase following initiation of pre-exposure prophylaxis (PrEP) for HIV prevention. We used longitudinal data from HIV-uninfected participants (n = 1013) enrolled in an open-label study of PrEP delivered to Ugandan and Kenyan heterosexual HIV serodiscordant couples to understand the association between PrEP initiation and HIV risk-related sexual behaviors among these couples. In the month following PrEP initiation, the mean number of monthly sex acts within couples decreased from 7.9 to 6.9 (mean difference: − 1.1; 95% CI − 1.5, − 0.7) and the proportion of couples having condomless sex decreased from 65% to 32% (percentage point change: − 33%; 95% CI − 37%, − 30%); these behaviors then remained relatively constant over 2 years. We found no evidence of sexual risk compensation following PrEP initiation within African serodiscordant couples. However, roughly a third of couples continued to engage in condomless sex during follow up, emphasizing the importance of continued PrEP use to sustain HIV protection.

## Introduction

Pre-exposure prophylaxis (PrEP) is highly effective at preventing HIV infection [1–4], but concerns remain that sexual risk compensation following PrEP initiation may be associated with increased incidence of sexually transmitted infections (STIs) or HIV (if PrEP adherence is low) due to higher rates of condomless sex and a higher number of sexual partners [5]. Risk compensation is a change in behavior in response to a change in perceived level of risk [6]. In the context of HIV prevention, one example of risk compensation is when individuals initiating PrEP increase HIV risk-related sexual behaviors because of their belief that PrEP protects them from infection.

There is emerging evidence that sexual risk compensation following PrEP initiation may occur among men who have sex with men (MSM) in high-income countries [7–11]. Some studies, primarily from cohorts of MSM on PrEP, have shown that PrEP initiation increases the frequency of condomless sex [7–9, 12, 13], number of sexual partners [8, 9, 11], and the incidence of STIs [10], among members of HIV at-risk populations. A previous study among heterosexual HIV serodiscordant couples, enrolled in an open-label extension to a PrEP efficacy trial in sub-Saharan Africa, the Partners PrEP Study, found no evidence of sexual risk compensation following PrEP initiation [14]. No known study to date has examined the effect of PrEP initiation on sexual behaviors associated with HIV risk among heterosexual HIV serodiscordant couples in the context of open-label PrEP use and counseling about the high efficacy of PrEP for HIV prevention.

The literature suggests that heterosexual HIV serodiscordant couples have strong incentives for increasing sexual behaviors associated with HIV risk following PrEP initiation [15–20]. First, serodiscordant couples have reported that knowledge of serodiscordance alienates them because they fear HIV transmission via condomless sex. Additionally, these couples reported that condom use works against re-establishing intimacy and closeness, further elongating their emotional distance [15, 16, 18]. Second, many couples report the desire for children and PrEP provides an opportunity to practice safer conception to minimize transmission risk between them [15, 17, 19, 20]. There is also evidence, however, that following PrEP initiation sexual behaviors associated with HIV risk may not change within HIV serodiscordant couples for reasons including inconsistent PrEP adherence—patterns that are often established early after initiation [5, 21], lack of trust in the protective effects of PrEP for HIV prevention [16], and complex partner dynamics surrounding the negotiation of sex and condom use [20, 22].

In this study, we aimed to understand the association between PrEP initiation and HIV risk-related sexual behaviors among heterosexual HIV serodiscordant couples accessing PrEP in a demonstration project in sub-Saharan Africa. While we refer to sexual behaviors as “HIV risk-related” in this study, it is important to note that these behaviors are only associated with increased HIV risk in the absence of all other HIV prevention measures including consistent PrEP use and sustained ART use by partners living with HIV. As PrEP becomes more available worldwide, a better understanding of sexual behaviors among heterosexual HIV serodiscordant couples following PrEP initiation in a real-word setting is important for the design of PrEP counseling interventions for couples.

## Methods

### Population and Procedures

From November 2012 to August 2014, participants were enrolled in the Partners Demonstration Project—a prospective, open-label implementation study that evaluated the delivery of daily oral PrEP integrated in existing antiretroviral treatment (ART) services among high-risk, heterosexual HIV serodiscordant couples in Kenya and Uganda [23, 24]. Four clinical care research sites in Thika and Kisumu, Kenya and in Kampala and Kabwohe, Uganda implemented the study. Couples were eligible for study participation if they were ≥ 18 years of age, sexually active, and planned to remain a couple for ≥ 1 year. Couples were excluded if the partner living with HIV was already using ART based on national clinical guidelines at the time (incorporating CD4 count and clinical conditions).

At enrollment, all couples were counseled on the HIV prevention benefits of early ART and PrEP, and all HIV-uninfected participants were offered PrEP, prescribed as daily co-formulated emtricitabine/tenofovir disoproxil fumarate (FTC/TDF). Couples were scheduled for return visits 1 month after enrollment, 2 months later, and quarterly thereafter. At each visit, HIV-negative partners were tested for HIV according to the national algorithm (a rapid HIV test, confirmed by a different rapid HIV test if positive, with a “tie-breaker” third test if the two rapid test results were inconsistent [25]), provided PrEP refills (as needed), and couples-based HIV prevention counseling including counseling about daily adherence for PrEP. PrEP discontinuation was recommended for the HIV-uninfected partner when the partner living with HIV had used ART for ≥ 6 months (expected to be commensurate with viral suppression) and there were no concerns by either member of the couples about ART adherence or additional sex partners, and the couple did not have immediate plans to become pregnant [23, 24].

### Data Collection

Couples were followed for up to 2 years and completed standardized questionnaires at each clinic visit to collect demographic, medical (including ART use by the partner living with HIV), and sexual behavior data. All data were captured on case report forms in face-to-face interviews by experienced quantitative researchers, using the participants’ preferred language (English, Kiswahili [Kenya], Dholuo [Kisumu], Kikuyu [Thika], Luganda [Kampala], Runyankore [Kabowhe]). The paper forms were then faxed and translated by intelligent character recognition software into electronic data via DataFax (DF/Net Research, Inc, Seattle, USA).

### Sexual Behavior Outcomes

We measured three sexual behavior outcomes in this study: (i) the number of sex acts with a study partner in the past month, (ii) any condomless sex with a study partner in the past month, and (iii) any sex with a non-study partner in the past month. Sex acts included either vaginal or anal sex. All sexual behavior outcomes were self-reported by the HIV-uninfected study partner. We categorized participants as engaging in any condomless sex if they reported fewer sex acts with a condom than the total number of sex acts with their study partner in the past month.

### PrEP Initiation

We measured PrEP initiation, captured on pharmacy records, two different ways for this study: (i) as a binary variable (initiation/no initiation), and (ii) a categorical variable, representing different time periods since initiation. We included six categories in our time since PrEP initiation variable: time prior to PrEP initiation (reference), within 1 month of post PrEP initiation, 1 to 3 months post PrEP initiation, 3 to 6 months post PrEP initiation, 6 to 12 months post PrEP initiation, and more than 12 months post PrEP initiation. We included the categorical measure of time since PrEP initiation to determine whether changes in HIV serodiscordant couples’ sexual behaviors varied with increasing time since PrEP initiation. Adherence to PrEP was measured by electronic medication event monitoring (MEMS) bottle caps given to each participant. MEMS caps recorded a date-time stamp each time the bottle was opened and data were downloaded at each participant visit. We categorized participants as PrEP adherent if they took > 80% of their expected PrEP doses, according to MEMS data [21].

### Time-Varying Covariates

At each clinic visit, we measured a number of other variables that are likely to change as time since study enrollment and PrEP initiation progresses, including participants’ pregnancy intentions (trying, not trying, pregnant), recent treatment for a genital infection, and use of hormonal contraception.

### Statistical Methods

We used linear probability models with individual fixed effects to measure the association between PrEP initiation and sexual behaviors: (i) number of sex acts with a study partner, (ii) any condomless sex with a study partner, and (iii) any sex with a non-study partner in the past month, as reported by the HIV-negative partner. For the number of sex acts outcomes (count variable), model coefficients are interpreted as mean differences in sex acts following PrEP initiation, while for the any condomless sex and any sex with a non-study partner outcomes (binary variables), coefficients are interpreted as the percentage point changes in the outcomes following PrEP initiation. Based on a priori decisions, we included pregnancy intentions, recent treatment for genital infection, use of hormonal contracetion, and sex with a non-study partner (for outcomes with a study partner only) as time-varying confounders in our models because these may be strongly associated with PrEP use and HIV risk-related sexual behaviors. We also included calendar month of observation in the models to adjust for other community-level changes, such as social marketing campaigns, health system reforms, or general underlying societal trends that may affect all participants. For the models measuring the association between PrEP initiation and any condomless sex, we included the same factors plus the number of sex acts in the past month to control for changes in study couples’ frequency of sex over time.

For these models, we limited the sample population to participants who reported sex with their study partner in the past month—a strong indicator that the couple is still together and PrEP use is warranted, and participants that had more than two PrEP clinic visits—so that differences in sexual behavior following PrEP initiation could be compared. Additionally, in all models, we censored participants at the first visit they reported not using PrEP.

### Sensitivity Analyses

We conducted three sensitivity analyses to test the findings from the main analyses. First, to minimize a potential increase in HIV risk-related sexual behaviors associated with perceived viral load suppression among the study partner living with HIV, we censored data for participants whose study partner had been on ART for over 6 months. Second, to isolate the association between PrEP initiation and sexual behaviors among participants who were adherent to PrEP and thus truly protected from HIV infection, we limited the sample to participants who were highly PrEP adherent (with ≥ 80% of expected doses taken based on MEMS data) following PrEP initiation. Third, to disentangle the association between participants’ recent knowledge of their study partners’ HIV-positive status from the association between PrEP initiation on HIV risk-related sexual behaviors, we limited the study sample to participants who learned their study partner was living with HIV more than 3 months prior to enrollment. We used the same individual fixed effects panel estimation described above for both sensitivity analyses.

### Sub-group Analyses

We conducted two sub-group analyses, where we measured the association between PrEP initiation and heterosexual HIV serodiscordant couples’ HIV risk-related sexual behaviors by the sex of the HIV-uninfected partner reporting the outcomes (e.g., male or female). It is well established that compared to men in sub-Saharan African settings, women have less ability to negotiate sex, including both the frequency at which sex occurs and condom use during sex [26, 27].

We used Stata 15.1 (Stata Corporation, College Station, Texas) to conduct all analyses.

## Results

From November 2012 to August 2014, 1694 HIV heterosexual serodiscordant couples were screened and 1013 were enrolled in the Partners Demonstration Project. Of these, 941 (93%) had an HIV-uninfected partner that initiated PrEP and met our definition for inclusion in this study sample.

At baseline, the median age of the HIV-uninfected participants included in our sample was 29 years (interquartile range [IQR] 26 to 36) (Table 1). The majority of participants were male (n = 635, 67%), married (n = 892, 95%), and living with their study partner (n = 914, 97%). Knowledge among participants of their study partners’ HIV-positive status was recent (median number of months known HIV serodiscordant: 1, IQR 1 to 3) and the majority of participants reported that they were not currently trying to get pregnant (n = 767, 82%). In the month prior to enrollment, the median number of sex acts between study partners was 6 (IQR 3 to 10), and the majority of participants reported at least one condomless sex act with their study partner (n = 632, 67%). Few participants reported any sex with a non-study partner in the past month (n = 78, 8%).

**Table 1:** Participant characteristics at baseline, n = 941

Age (med, IQR)	29 (26 to 36)
Sex	
Male	634 (67%)
Female	307 (33%)
Education	
No formal (0 years)	38 (4%)
Primary (1–8 years)	492 (52%)
Secondary (9–12 years)	270 (29%)
Tertiary (> 12 years)	141 (15%)
Monthly income, USD^a^	
No income	132 (14%)
0 to < $25	214 (22%)
$25 to < $50	151 (16%)
$50 to < $100	100 (21%)
> $100	245 (26%)
Married to study partner	892 (95%)
Living with study partner	913 (97%)
Years living with study partner (med, IQR)	2.5 (0.8 to 6.7)
Months known of partner’s HIV-positive status	
< 1 month	201 (21%)
1 month	436 (46%)
2 months	45 (5%)
> 3 months	259 (26%)
Viral load of study partner living with HIV, copies/mL (med, IQR)	37,240 (7512 to 104,598)
Using a hormonal family planning method^b^	102 (11%)
Ever treated for a genital tract infection	22 (2%)
Pregnancy desires	
Not pregnant, not trying	766 (81%)
Not pregnant, trying	62 (7%)
Pregnant	112 (12%)
Number of sex acts with partner, past month (med, IQR)	6 (3 to 10)
Any condomless sex with study partner,^c^ past month	631 (67%)
Any sex with a non-study partner, past month	78 (8%)

*med* median, *IQR* interquartile range, *SD* standard deviation, *ART* antiretroviral treatment

^a^Converted Kenya and Uganda Shilling to US dollars using the going rates for October 3, 2013 (Kenya: 86.8 KES to 1 USD; Uganda: 2556.4 UGX to 1 USD)

^b^Includes implants, injectable, IUD, and oral contraception pills

^c^Participant reports having condomless sex with study partner at least once in the past month

**Fig. 1:**
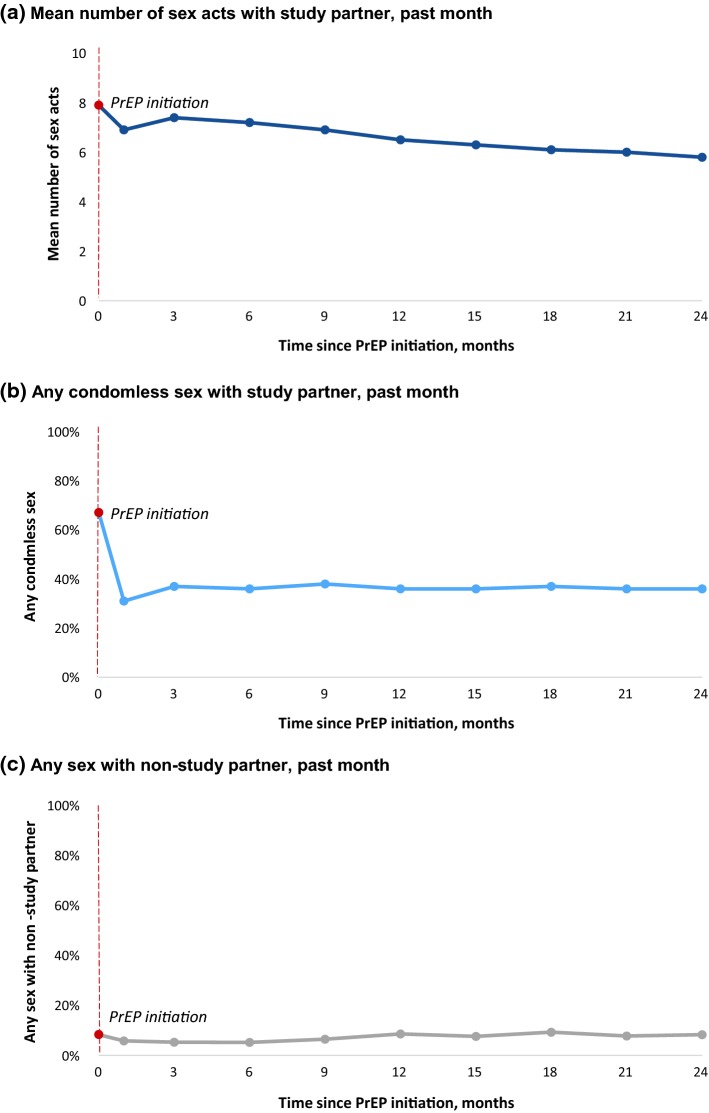
Heterosexual HIV serodiscordant couples’ sexual behaviors following PrEP initiation

Over the 2-year study period, participants’ sexual behaviors remained relatively constant following PrEP initiation (among those reporting sex) (Fig. 1). In the first month following PrEP initiation, both the mean number of sex acts and any condomless sex between study partners in the past month were reduced, but then remained relatively stable over time. The prevalence of any sex with a non-study partner remained low and stable over time.

**Fig. 2:**
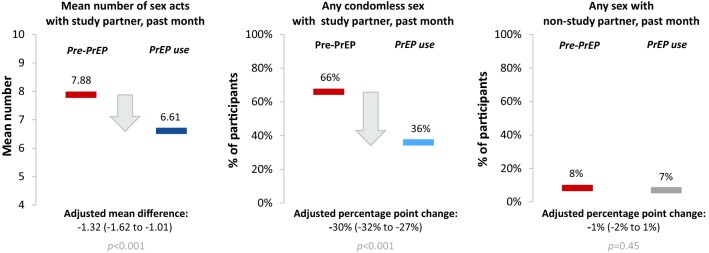
The associations between PrEP initiation and HIV risk-related sexual behaviors among heterosexual HIV serodiscordant couples. Associations measured using individual longitudinal data and linear regression models with fixed effects for individuals, fertility intentions (trying, not trying, pregnant), sex with a non-study partner (study partner outcomes only), treatment for a genital infection, use of hormonal contraception, calendar month, and number of sex acts (any condomless sex outcome only)

Following PrEP initiation, couples decreased their mean number of sex acts within the couple from 7.88 to 6.61 acts (adjusted mean difference: − 1.32, 95% confidence interval [CI] − 1.62 to − 1.01, *p *< 0.001) and the prevalence of any condomless sex within the couple from 66% to 36% (adjusted percentage point change: − 30%, 95% CI − 32% to − 27%, *p *< 0.001) over the 2 year follow-up period (Fig. 2). There were no statistically significant differences in the prevalence of any sex with a non-study partner following PrEP initiation (adjusted percentage point change: − 1%, 95% CI − 2% to 1%, p = 0.45) over the study period.

**Fig. 3:**
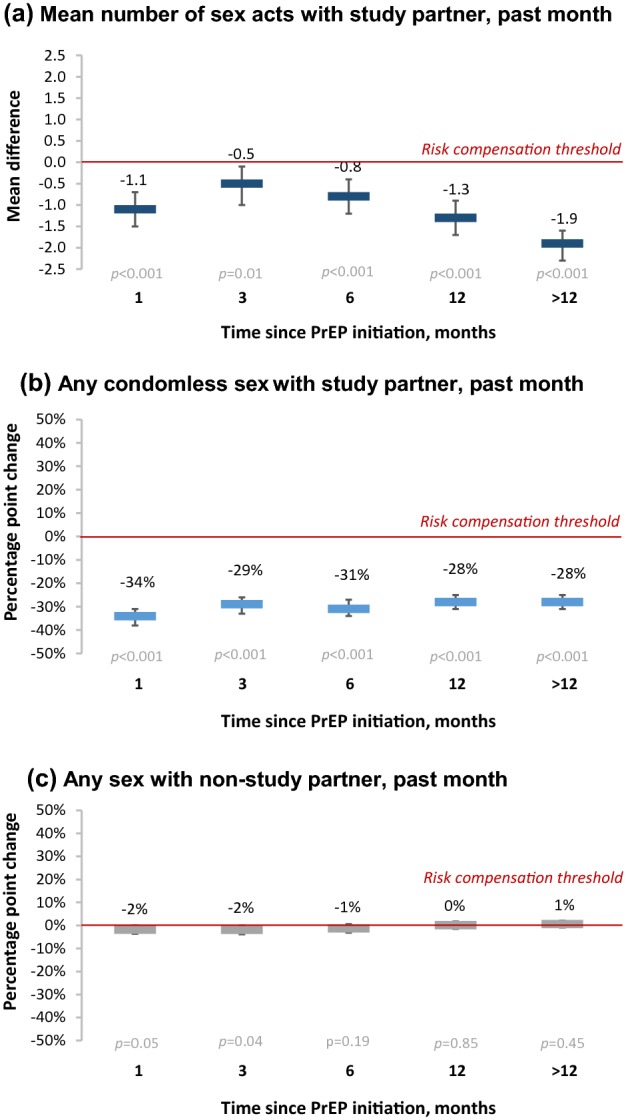
The associations between time since PrEP initiation and HIV risk-related sexual behaviors within heterosexual HIV serodiscordant couples. Associations measured using individual longitudinal data and linear regression models with fixed effects for individuals, fertility intentions (trying, not trying, pregnant), sex with a non-study partner (study partner outcomes only), treatment for a genital infection, use of hormonal contraception, calendar month, and number of sex acts (any condomless sex outcome only). Associations above the red line would suggest sexual risk compensation—i.e., an increase in HIV risk-related sexual behaviors within serodiscordant relationships following PrEP initiation

The statistically significant declines in HIV risk-related sexual behaviors within HIV serodiscordant couples remained consistent at all time periods following PrEP initiation (Fig. 3). The prevalence of any sex with a non-study partner declined slightly in the first (percentage point change: − 2%, 95% CI − 4% to 0%, p = 0.05) and third month (percentage point change: − 2%, 95% CI − 4% to 0%, p = 0.04) following PrEP initiation, then there were no significant changes at the other time periods.

In our sensitivity analyses, these findings remained consistent among (i) participants whose study partner living with HIV had been on ART for ≤ 6 months, and (ii) participants who were adherent to PrEP, but not among (iii) participants who knew their study partner was living with HIV for ≥ 3 months prior to enrollment (Appendix Table 3). In the last sensitivity analysis, there were few statistically significant decreases in HIV risk-related sexual behaviors within study couples following PrEP initiation, and the magnitude of the decreases that were statistically significant were small.

In the sub-group analyses, which explored the associations between PrEP initiation and HIV risk-related sexual behaviors by sex of the HIV-uninfected couple, the findings from the main analyses were consistent, but the magnitudes of the associations measured were greater among male participants (Table 2). For example, the mean number of sex acts within study partners following PrEP initiation decreased by − 1.59 acts (95% CI − 2.00 to − 1.19, *p *< 0.001) among males participants versus − 0.73 acts (95% CI − 1.19 to − 0.27, *p *= 0.004) among female participants, and the sex-based differences were statistically significant (interaction *p *= 0.008). The prevalence of any condomless sex within study partners following PrEP initiation deceased by − 37% (95% CI − 41% to − 33%, *p *< 0.001) among male participants in the first month and by − 30% (95% CI − 36% to − 23%, *p *< 0.001) among female participants in the first month (interaction *p *= 0.02). The greater associations between PrEP initiation and HIV risk-related sexual behaviors for male versus female participants remained consistent at all periods of time following PrEP initiation (e.g., 1 month, 1 to 3 months, 3 to 6 months, 6 to 12 months, 12 to 24 months).

**Table 2:** Sub-group analyses: the associations between PrEP initiation and HIV risk-related sexual behaviors, by sex of the HIV-uninfected partner

	Males, n = 635		Females, n = 307		Interaction *p*
	MD/PP (95% CI)	*p*	MD/PP (95% CI)	*p*	
Mean number of sex acts with study partner, past month (MD)					
PrEP initiation	− 1.59 (− 2.00 to − 1.19)	< 0.001	− 0.73 (− 1.19 to − 0.27)	0.002	0.008
Time since PrEP initiation					
1-month	− 1.45 (− 1.99 to − 0.92)	< 0.001	− 0.47 (− 1.08 to 0.14)	0.13	0.73
3-months	− 0.62 (− 1.18 to − 0.06)	0.03	− 0.37 (− 0.99 to 0.25)	0.24	0.03
6-months	− 1.17 (− 1.74 to − 0.59)	< 0.001	− 0.03 (− 0.67 to 0.60)	0.92	0.88
12-months	− 1.53 (− 2.03 to − 1.04)	< 0.001	− 0.80 (− 1.36 to − 0.25)	0.004	0.16
> 12-months	− 2.29 (− 2.74 to − 1.83)	< 0.001	− 1.16 (− 1.66 to − 0.65)	< 0.001	–
Any condomless sex^a^ with study partner, past month (PP)					
PrEP initiation	− 32% (− 35% to − 28%)	< 0.001	− 27% (− 32% to − 22%)	< 0.001	0.08
Time since PrEP initiation					
1-month	− 37% (− 41% to − 33%)	< 0.001	− 30% (− 36% to − 23%)	< 0.001	0.02
3-months	− 33% (− 38% to − 29%)	< 0.001	− 22% (− 28% to − 16%)	< 0.001	< 0.001
6-months	− 32% (− 37% to − 28%)	< 0.001	− 29% (− 35% to − 22%)	< 0.001	0.14
12-months	− 29% (− 33% to − 25%)	< 0.001	− 26% (− 32% to − 20%)	< 0.001	0.14
> 12-months	− 29% (− 32% to − 25%)	< 0.001	− 29% (− 34% to − 23%)	< 0.001	–
Any sex with non-study partner, past month (PP)					
PrEP initiation	− 1% (− 3% to 1%)	0.18	1% (0% to 3%)	0.10	0.07
Time since PrEP initiation					
1-month	− 2% (− 5% to 0%)	0.09	− 1% (− 3% to 1%)	0.37	0.51
3-months	− 3% (− 6% to 1%)	0.02	1% (− 1% to 3%)	0.52	0.34
6-months	− 2% (− 5% to 0%)	0.10	1% (− 1% to 3%)	0.38	0.66
12-months	− 1% (− 3% to 2%)	0.62	2% (0% to 4%)	0.03	0.95
> 12-months	0% (− 2% to 2%)	0.92	2% (1% to 4%)	0.01	–

Associations measured using individual longitudinal data and linear regression models with fixed effects for individuals, fertility intentions (trying, not trying, pregnant), sex with a non-study partner (study partner outcomes only), treatment for a genital infection, use of hormonal contraception, calendar month, and number of sex acts (any condomless sex outcome only)

*MD* mean difference, *CI* confidence interval, *P p* value, *n* number of participants, *PP* percentage point change

^a^Participant reports having condomless sex with study partner at least once in the past month

## Discussion

We found no evidence of sexual risk compensation among stable, heterosexual HIV serodiscordant couples in Kenya and Uganda following PrEP initiation. In fact, HIV serodiscordant couples in these settings appeared to modestly decrease HIV risk-related sexual behaviors within the couple in the month following PrEP initiation, and then maintain these lower levels of HIV risk-related sexual behaviors with the couple over time. This finding is different than studies among MSM in high-income countries, which have observed sexual risk compensation following PrEP initiation [7–11]. It is consistent, however, with the study among heterosexual HIV serodiscordant couples enrolled in an open-label extension to a PrEP efficacy trial, which found no evidence of sexual risk compensation following PrEP initiation in Kenya and Uganda [14]. Even though we observed no evidence of sexual risk compensation among serodiscordant couples in this study, many couples continued to engage in condomless sex within the couple throughout the 2-year study period, emphasizing the important prevention gap filled by PrEP.

The observed decrease in HIV risk-related sexual behaviors within heterosexual HIV serodiscordant couples in the month following PrEP initiation may be attributable to the counseling couples received when initiating PrEP. While PrEP is nearly 100% effective at preventing HIV infection when adherence is high, the counseling delivered with PrEP in the Partners Demonstration Project still emphasized the importance of continued condom use to prevent pregnancy, STIs, and as a backup for HIV prevention [23, 24]. HIV serodiscordant couples have reported the use of condoms as a barrier to establishing intimacy and closeness within the relationship [15, 16, 20]. Thus, counseling on continued condom use for HIV prevention with the delivery of PrEP may be a missed opportunity to help HIV serodiscordant couples re-establish intimacy in their relationship. The use of condoms for HIV protection has been engrained in HIV prevention counseling for the past three decades [28–30], thus more research would enable better understanding of how this message could be appropriately modified to best suit the needs of HIV serodiscordant couples on PrEP, especially as the partner living with HIV achieves suppressed HIV viremia with consistent ART use and thus is no longer infectious [31].

The majority of participants in this study learned their study partner was living with HIV a month or less than a month before they initiated PrEP, which may have also contributed to the observed decrease in HIV risk-related sexual behaviors within heterosexual HIV serodiscordant couples in the month following PrEP initiation. HIV serodiscordant couples face a fragile period in their relationship when they learn their discordant diagnosis and often change their sexual behaviors in response to this knowledge [32, 33]. In this study, it was difficult for us to disentangle the association between knowledge among participants of their study partner’s HIV-positive status on HIV risk-related sexual behaviors from that of PrEP initiation on HIV risk-related sexual behaviors because often these two events happened very close to one another. This was the motivation for our third sensitivity analysis, limited to couples who knew their discordant status > 3 months prior to study enrollment, which continued to find no evidence of sexual risk compensation although a smaller magnitude of decreasing HIV-related sexual behavior. This suggests that PrEP initiation alone is likely not associated with sexual risk compensation and that recent knowledge among participants of their study partner’s HIV-positive status may be driving the large decreases in HIV risk-related sexual behaviors following PrEP initiation in this study.

This study has a number of strengths. First, it is evaluating sexual risk compensation following PrEP initiation in the real-word context of open-label PrEP use and counseling about the high efficacy of PrEP for HIV prevention. Second, it is doing so among a large population of heterosexual HIV serodiscordant couples—a key population for HIV prevention interventions for which there is limited literature on risk compensation and PrEP [14]—in two sub-Saharan African settings. Third, the individual fixed effects estimation approach is a rigorous quasi-experimental method for causal inference, that controls for all individual-level baseline and other time-invariant characteristics, including those that are unobserved (e.g., psychological characteristics that are likely to sexual behaviors) and thus cannot be controlled for as covariates in our analyses [34, 35].

This study also has limitations. First, all sexual behavior outcomes within couples were self-reported by participants and thus subject to social desirability and recall bias, which may have resulted in an overestimation of the decreases in HIV risk-related sexual behaviors within couples following PrEP initiation. Second, at the time of the study, PrEP was still a new HIV prevention intervention and not available to heterosexual HIV serodiscordant couples in Kenya and Uganda except through the study. This unfamiliarity with PrEP for HIV prevention might have made participants unsure of the effectiveness of the new technology, and thus participants may not have been motivated to change their HIV risk-related sexual behaviors following PrEP initiation. Third, we only have measurements of couples’ sexual behaviors prior to PrEP initiation at baseline. Thus, our sexual behavior measurements in the pre-PrEP period may not reflect the couples’ regular sexual behaviors and could be subject to monthly fluctuations in sexual practices. Fourth, sexual behaviors within couples are likely to change over the duration of relationships, and thus we may be capturing some of these changes in our analyses. In particular, the frequency of sex within couples is likely to decrease over time as relationships become more established [36] or increase during periods when couples are trying to conceive. Finally, since the majority of participants learned of their partner’s HIV-positive status around the same time that they initiated PrEP, it was difficult to disentangle the association between this recent knowledge and HIV risk-related sexual behaviors from that of PrEP initiation and HIV risk-related sexual behaviors.

The generalizability of our study results are also limited. First, this study focused on measuring sexual risk compensation following PrEP initiation within heterosexual HIV serodiscordant couples that planned to stay in a relationship with one another and reported behaviors suggesting that they were largely stable. Thus, our findings cannot be generalized to sexual behaviors of other couples in this setting that might have more dynamic partnerships and less relationship stability. Second, there are significant cultural variations throughout sub-Saharan Africa in regard to heterosexual relationships and the negotiation of sex within these relationships. In our sub-group analysis that looked at changes in HIV risk-related sexual behaviors by sex of the HIV-uninfected partner, we saw greater decreases in HIV risk-related sexual behaviors following PrEP initiation when the HIV-uninfected partner was male, suggesting that in these contexts, men have more sway within heterosexual relationships on sexual frequency and condom use. The negotiation of sex within heterosexual couples might vary in other cultural contexts outside of Kenya and Uganda, resulting in different findings.

## Conclusions

Governments in sub-Saharan African countries considering including or expanding the delivery of PrEP within their national HIV prevention programs can be relieved of concerns about sexual risk compensation following PrEP initiation among stable, heterosexual HIV serodiscordant couples. Despite counseling in this study on the importance of continued condom use following PrEP initiation, condomless sex within study couples remained high—emphasizing importance of good PrEP adherence for HIV prevention and the need for PrEP to provide HIV prevention prior to ART use and viral suppression. When delivering PrEP to couples who recently have learned of their HIV serodiscordant status, PrEP can be part of a comprehensive HIV prevention package that includes both adherence and risk reduction counseling. To promote the use of PrEP among serodiscordant couples, this counseling might actually want to emphasize the role of condomless sex in the context of high PrEP adherence, contraception, and STI testing and treatment, in order to promote re-establishment of intimacy as they wait for the partner living with HIV to start ART and achieve viral suppression.

## Appendix

See Table 3.

**Table 3:** Sensitivity analyses: the associations between of PrEP initiation and HIV risk-related sexual behaviors within heterosexual serodiscordant couples

	Mean number of sex acts with study partner, past month		Any condomless sex^a^ with study partner, past month		Any sex with non-study partner, past month	
	MD (95% CI)	*p*	PP (95% CI)	*p*	PP (95% CI)	*p*
1. Censor participants when the study partner living with HIV has been on ART for 6 mos, n = 941						
PrEP initiation	− 1.10 (− 1.46 to − 0.75)	< 0.001	− 32% (− 35% to − 29%)	< 0.001	− 1% (− 3% to 0%)	0.04
Time since PrEP initiation						
1-month	− 1.20 (− 1.65 to − 0.75)	< 0.001	− 34% (− 38% to − 31%)	<0.001	− 2% (− 3% to 0%)	0.03
3-months	− 0.65 (− 1.11 to − 0.18)	0.01	− 29% (− 33% to − 25%)	< 0.001	− 2% (− 4% to 0%)	0.03
6-months	− 0.96 (−1.50 to − 0.42)	0.001	− 30% (− 35% to − 26%)	< 0.001	− 2% (− 4% to 1%)	0.14
12-months	− 1.66 (− 2.22 to − 1.10)	< 0.001	− 31% (− 35% to − 26%)	< 0.001	1% (− 1% to 3%)	0.55
>12-months	− 1.87 (− 2.60 to − 1.15)	< 0.001	− 35% (−41% to − 29%)	< 0.001	− 1% (−3% to 2%)	0.66
2. Censor participants not adhering to PrEP^b^, n = 940						
PrEP initiation	− 0.99 (− 1.38 to − 0.60)	< 0.001	− 31% (− 34% to − 27%)	<0.001	− 1% (− 2% to 0%)	0.22
Time since PrEP initiation						
1-month	− 1.17 (− 1.66 to − 0.68)	< 0.001	− 35% (− 39% to − 31%)	< 0.001	− 1% (− 3% to 1%)	0.21
3-months	− 0.38 (− 0.91 to 0.15)	0.16	− 30% (− 34% to -25%)	< 0.001	− 1% (− 3% to 0%)	0.11
6-months	− 0.46 (− 1.02 to 0.11)	0.11	− 30% (− 35% to − 26%)	< 0.001	− 1% (− 2% to 2%)	0.60
12-months	− 1.12 (− 1.64 to − 0.60)	< 0.001	− 26% (− 30% to − 21%)	< 0.001	− 1% (− 2% to 1%)	0.55
>12-months	− 2.03 (− 2.63 to − 1.44)	< 0.001	− 29% (− 34% to − 24%)	<0.001	0% (− 2% to 2%)	0.97
3. Limit to participants who knew their partner was living with HIV > 3 mos prior enrollment, n = 231						
PrEP initiation	− 0.44 (− 0.96 to 0.08)	0.10	− 6% (− 11% to − 1%)	0.02	0% (− 3% to 2%)	0.94
Time since PrEP initiation						
1-month	− 0.68 (− 1.38 to 0.03)	0.06	− 9% (− 16% to − 2%)	0.008	− 2% (− 5% to 1%)	0.22
3-months	− 0.19 (− 0.90 to 0.53)	0.61	− 6% (− 13% to 1%)	0.12	− 3% (− 7% to 0%)	0.05
6-months	0.27 (− 0.44 to 0.99)	0.45	− 6% (− 13% to 1%)	0.10	− 2% (− 5% to 2%)	0.30
12-months	− 0.21 (− 0.84 to 0.41)	0.50	− 6% (− 12% to 0%)	0.05	1% (− 2% to 4%)	0.58
>12-months	− 0.78 (− 1.34 to − 0.21)	0.01	− 5% (− 11% to 0%)	0.07	2% (− 1% to 5%)	0.19

Associations measured using individual longitudinal data and linear regression models with fixed effects for individuals, fertility intentions (trying, not trying, pregnant), sex with a non-study partner (study partner outcomes only), treatment for a genital infection, use of hormonal contraception, calendar month, and number of sex acts (any condomless sex outcome only)

*MD* mean difference, *CI* confidence interval, *P* p-value, *PP* percentage point change

^a^Participant reports having condomless sex with study partner at least once in the past month

^b^Participants took > 80% of expected doses, measured using electronic medication even monitoring (MEMS) bottle caps
